# Clinical signs, profound acidemia, hypoglycemia, and hypernatremia are predictive of mortality in 1,400 critically ill neonatal calves with diarrhea

**DOI:** 10.1371/journal.pone.0182938

**Published:** 2017-08-17

**Authors:** Florian M. Trefz, Ingrid Lorenz, Annette Lorch, Peter D. Constable

**Affiliations:** 1 Clinic for Ruminants with Ambulatory and Herd Health Services at the Center of Veterinary Clinical Medicine, LMU Munich, Oberschleißheim, Germany; 2 Bavarian Animal Health Service (Tiergesundheitsdienst Bayern e.V.), Poing, Germany; 3 College of Veterinary Medicine, University of Illinois, Urbana-Champaign, Illinois, United States of America; INIA, SPAIN

## Abstract

Profound acidemia impairs cellular and organ function and consequently should be associated with an increased risk of mortality in critically ill humans and animals. Neonatal diarrhea in calves can result in potentially serious metabolic derangements including profound acidemia due to strong ion (metabolic) acidosis, hyper-D-lactatemia, hyper-L-lactatemia, azotemia, hypoglycemia, hyperkalemia and hyponatremia. The aim of this retrospective study was to assess the prognostic relevance of clinical and laboratory findings in 1,400 critically ill neonatal calves with diarrhea admitted to a veterinary teaching hospital. The mortality rate was 22%. Classification tree analysis indicated that mortality was associated with clinical signs of neurologic disease, abdominal emergencies, cachexia, orthopedic problems such as septic arthritis, and profound acidemia (jugular venous blood pH < 6.85). When exclusively considering laboratory parameters, classification tree analysis identified plasma glucose concentrations < 3.2 mmol/L, plasma sodium concentrations ≥ 151 mmol/L, serum GGT activity < 31 U/L and a thrombocyte count < 535 G/L as predictors of mortality. However, multivariable logistic regression models based on these laboratory parameters did not have a sufficiently high enough sensitivity (59%) and specificity (79%) to reliably predict treatment outcome. The sensitivity and specificity of jugular venous blood pH < 6.85 were 11% and 97%, respectively, for predicting non-survival in this study population. We conclude that laboratory values (except jugular venous blood pH < 6.85) are of limited value for predicting outcome in critically ill neonatal calves with diarrhea. In contrast, the presence of specific clinical abnormalities provides valuable prognostic information.

## Introduction

Severe acidemia due to strong ion (metabolic) acidosis is associated with an increased mortality rate in critically ill human patients and is frequently associated with hyper-L-lactatemia secondary to sepsis, hypovolemic shock, hepatic failure, and vascular injury [1–6]. Hemoconcentration, azotemia, hypoglycemia, hyponatremia, hyperkalemia, septicemia, hyper-D-lactatemia, hyper-L-lactatemia, and the development of a strong ion (metabolic) acidosis are well known complications of neonatal diarrhea in calves [7–10]. An increase in the plasma concentration of D-lactate, L-lactate, and other unmeasured strong anions can be quantified by calculating the anion gap or strong ion gap [7–10]. Consequently, acid-base and serum biochemical variables might provide clinically useful prognostic indicators in critically ill calves with diarrhea. Previous studies using small study populations of diarrheic calves identified hemoconcentration and increased serum urea, potassium and chloride concentrations as risk factors for death [11, 12], but these studies did not specifically investigate the prognostic value of acid-base values such as blood pH or the plasma concentrations of glucose, D-lactate, and L-lactate, or the prognostic value of clinical findings.

Critically ill calves with diarrhea typically exhibit variable degrees of dehydration, depression, decreased or loss of the suckling reflex, and impaired ability to stand [13–16]. The neurological signs expressed by diarrheic calves are primarily due to increased plasma concentrations of D-lactate that is absorbed from the gastrointestinal tract following bacterial fermentation of ingested milk [14, 17, 18]. Hyper-D-lactatemia has also been reported in neonatal lambs and kids [19, 20]. Hyper L-lactatemia plays a quantitatively unimportant role in the development of strong ion acidosis in diarrheic calves, although moderate increases in plasma L-lactate concentrations occurs in calves with septicemia or marked hypovolemia and concomitant tissue hypoperfusion [8, 9, 21]. In critically ill neonatal foals admitted to intensive care units, increased plasma L-lactate concentration was associated with bacteremia and evidence of the systemic inflammatory response syndrome (SIRS); hyper L-lactatemia therefore provided a reliable indicator of non-survival in these animals [22, 23]. Similar to hyper L-lactatemia, hypoglycemia has been associated with sepsis and a low survival rate in neonatal calves and foals [24, 25].

Diarrheal diseases account for 15% of the deaths in children under five years of age worldwide [26], with death being more likely to occur in diarrheic children with co-morbidities such as pneumonia or malnutrition [27, 28]. The prognostic value of decreased blood pH, hyper-L-lactatemia, hyper-D-lactatemia, and increased anion gap (AG) in children under five with diarrhea has not been extensively investigated. Hyper-D-lactatemia is a complication of the short-bowel syndrome in humans following resection of large parts of the small intestine and the cause of a high AG metabolic acidosis in affected patients [29]. A high AG metabolic acidosis has also been described in diarrheic children and infants [30, 31]. The presence of severe malnutrition, clinical dehydration, sepsis, pneumonia or respiratory distress, drowsiness, meningitis, abdominal distention, and absent peripheral pulses, as well as hypoglycemia, hypoxemia, hypoalbuminemia, hyponatremia and hypernatremia, have been identified as independent predictors of death in diarrheic children [28, 32–35]. These results suggest that the prognostic accuracy can be improved in diarrheic children when clinical signs are evaluated in combination with laboratory values, instead of basing prognosis only on the results of laboratory analysis.

In spite of numerous and potentially serious metabolic derangements in neonatal diarrheic calves, previous studies that were based on referral hospital populations reported relatively high survival rates ranging from 70 to 80% [8, 36, 37]. However, because of animal welfare concerns and the cost of treatment in severely affected calves, it would be desirable to identify clinically useful prognostic factors that reliably predict the treatment outcome. The aim of this study was therefore to use descriptive statistics (decile comparisons, univariate methods), and classification tree analysis followed by multivariate logistic regression to identify risk factors for non-survival in a large study population of hospitalized critically ill neonatal calves with diarrhea. Specifically, we were interested in characterizing the association between treatment outcome and specific clinical findings as well as acid-base indices and the results of serum and plasma biochemical analysis (including plasma D-lactate, L-lactate, and glucose concentrations). We anticipated that the results of this study would identify clinically important factors related to death in critically ill neonatal animals, and that the results of the study would assist in identifying independent prognostic factors in children under five years of age with diarrhea.

## Materials and methods

### Calves

A retrospective analysis was conducted utilizing the medical records of 1,400 diarrheic calves up to 21 days of age admitted to the Clinic for Ruminants, LMU Munich (Oberschleißheim, Germany) between April 2005 and January 2008, as well as between September 2009 and October 2012. Medical records were identified using the electronic database of the clinic and selected for inclusion in the study if calves were admitted to the clinic for treatment of diarrhea or had a clinical diagnosis of neonatal diarrhea on initial examination. Diarrhea was defined as a fecal consistency that permitted feces to run through slightly opened fingers. Information about the calf’s age, acid-base status and the results of serum biochemical analysis including glucose, D- and L-lactate, urea, creatinine, total protein, and phosphorus concentrations must have been obtained before treatment was administered.

### Review of medical records

Information retrieved from the medical records included signalment (age, sex, breed), rectal temperature, heart rate, respiratory rate, presence or absence of injected scleral blood vessels and hyperemia of the mucous membranes, and the results of venous blood gas analysis, hematologic analysis, and serum biochemical analysis. The following clinical parameters were categorized using 3-point scales on the initial examination at admission: posture (ability to stand, impaired ability to stand, sternal or lateral recumbency), behavior (bright and alert, depressed, apathetic to comatose), suckling reflex (strong, weak, absent), degree of enophthalmos (none, slight to moderate, severe), and body condition (good to moderate, bad, cachectic). Concurrent health problems, including navel infections, bronchopneumonia, abdominal emergencies, neurologic (e.g. opisthotonus or seizures) or orthopedic abnormalities documented during the first 48 hours of hospitalization were also considered. Information regarding intraoperative and post mortem findings, and the stated reason for euthanasia, was also extracted from the medical record.

### Laboratory variables

Laboratory values were determined from blood samples routinely taken from the jugular vein for diagnostic purposes on admission. Lithium-heparinized blood samples were anaerobically collected using a 2-mL polypropylene syringe, and blood pH, partial pressure of carbon dioxide (pCO_2_) and oxygen (pO_2_), sodium, chloride and potassium concentrations determined using a blood pH, gas, and electrolyte analyzer (Rapidlab 865, Bayer Vital GmbH, Fernwald, Germany). Blood pH, pCO_2,_ and pO_2_ were corrected for rectal temperature using standard algorithms [38].

Automatic analyzers were used for hematologic analysis (Sysmex F-820 and pocH-100iV Diff, Sysmex Corporation, Kobe, Japan) and serum biochemical analysis (Hitachi 911 and 912, Roche Diagnostics, Indianapolis, USA). Hematologic parameters were determined using blood samples collected into EDTA containing tubes. Serum samples (plain tubes) were assayed for concentrations of urea (urease), creatinine (picric acid), total protein (biuret), albumin (bromcresol green), and inorganic phosphorus (ammonium molybdate) and activities of y-glutamyltransferase (L-y-glutamyl-3-carboxy-4-nitroanilid and glycylglycine), creatine kinase (creatinephosphate), aspartate aminotransferase (L-aspartate and 2-oxoglutarate) and glutamate dehydrogenase (α-ketoglutarate). Blood samples containing lithium heparin and potassium fluoride as a glycostatic agent were analyzed for plasma concentrations of D-lactate (D-lactate dehydrogenase), L-lactate (L-lactate dehydrogenase) [39, 40], and glucose (hexokinase).

### Calculations and definitions

Actual bicarbonate concentration was calculated by the blood gas unit using the Henderson-Hasselbalch equation with measured blood pH and pCO_2_ at 37°C:

$$cHCO_{3}^{-}=S\;\times \;pCO_{2}\;\times \;10^{\left(pH-pK1´\right)}$$ (1)

Values for the negative logarithm of the dissociation constant of carbonic acid (pK_1_^´^) and solubility of carbon dioxide (*S*) for plasma were 6.105 and 0.0307 mmol/L per mm Hg, respectively. After measuring the hemoglobin concentration (Hb; g/dL), blood base excess (in vitro base excess, mmol/L) was calculated using the van Slyke equation [41] with measured blood pH at 37°C and the calculated actual bicarbonate concentration:

$$Base\;excess=\left(1\;-\;0.014\;\times \;cHb\right)\;\times \;\left[\left(cHCO_{3}^{-}\;-\;24.8\right)\;+\;\left(1.43\;\times \;cHb\;+\;7.7\right)\;\times \;\left(pH\;-\;7.4\right)\right]$$ (2)

An estimate of the unmeasured anion concentration was obtained by calculating the anion gap (AG, mEq/L):

$$AG=\;cNa^{+}+\;cK^{+}\;-\;cCl^{-}\;-\;cHCO_{3}^{-}$$ (3)

In addition to the traditional Henderson-Hasselbalch model, the simplified quantitative physicochemical strong ion approach [42] was used in order to allow a more comprehensive assessment of the acid-base status of the calves. The concentration of nonvolatile weak acids (A_tot_, mmol/L) was calculated from the serum concentrations of total protein (g/L) according to experimentally determined values for calf plasma [7]:

$$A_{tot}=\;0.343\;\times \;c\left(total\;protein\right)$$ (4)

Measured strong ion difference was calculated from plasma concentrations of sodium, chloride, potassium, D-lactate and L-Lactate (SID_m_, mEq/L), such that:

$$SID_{m}=\;cNa^{+}+\;cK^{+}\;-\;cCl^{-}\;-\;c(D\;-\;lactate^{-})\;-\;c(L\;-\;lactate^{-})$$ (5)

An estimate of the unmeasured strong ion concentration was obtained by calculating the strong ion gap (SIG, mEq/L), which is defined as the difference between the plasma concentration of unmeasured strong cations and unmeasured strong anions [43]. This was performed using the calculated value for A_tot_, the experimentally determined value for the negative logarithm of dissociation constant of plasma nonvolatile weak acids (pKa) in calf plasma (pKa = 7.08) and the following equation [7]:

$$SIG\;=\;\left[A_{tot}\;/\;\left(1\;+\;10^{\left(7.08-pH\right)}\right)\right]\;-\;AG$$ (6)

The first expression on the right hand side of the equation represents the net negative charge of nonvolatile weak acids in plasma (A^-^, mEq/L). For the present analysis, SIG was corrected for measured plasma concentration for D- and L-lactate in order to obtain an estimate of the concentration of still unidentified strong ions (USI, mEq/L) which presented the unmeasured strong ion difference (SID_um_, mEq/L) such that:

$$USI\;=\;SID_{um}\;=\;SIG\;+\;c(D\;-\;lactate^{-})\;+\;c(L\;-\;lactate^{-})$$ (7)

A diagnosis of SIRS was made on admission using previously published definitions for SIRS in humans [44, 45] and established reference intervals for calves [46, 47] if two of the following criteria were fulfilled: presence of an abnormal leukocyte count (i.e. leukopenia or leukocytosis; reference interval, 5–12 G/L), abnormal rectal temperature (reference interval; 38.5–39.5°C), tachycardia (> 120 beats per minute), and tachypnea (> 36 breaths per minute). Calves were categorized as septicemic if they met the criteria for SIRS and if septicemia was clinically suspected based on the presence of marked hyperemia of mucous membranes, congestion/injection of episcleral vessels, mucosal or subscleral bleedings, or the presence of hypopyon. The probability of septicemia was also calculated using a published regression model on age, recumbency, absence of the suckling reflex, and presence of a focal infection (defined as hypopyon/uveitis, joint effusion, omphalitis, or neurologic symptoms suggestive of meningitis) [16]. Based on a reported sensitivity of 69% and a specificity of 75%, calves were considered septicemic for the present analysis if the calculated probability was ≥ 0.3 for the regression model [16].

### Treatment of calves

Calves were treated with oral electrolyte solutions and if indicated with constant drip infusions consisting of sodium bicarbonate, saline, and glucose solutions. Intravenous fluid therapy was continued until clinical signs of metabolic acidosis or dehydration had normalized and calves were able to counterbalance the enteral fluid and electrolyte losses by the oral intake of milk and an electrolyte solution. Additional supportive therapy consisted of the parenteral administration of nonsteroidal drugs and the supplementation of vitamin E and selenium. Antimicrobial therapy was initiated in cases of a concurrent bacterial infection, suspected septicemia, fever, or the presence of hypothermia on admission to the hospital. Surgical interventions due to complicated navel infections, umbilical herniation, or suspected abdominal emergencies were performed in 109 calves. Relatively uniform treatment procedures were ensured by daily patient rounds performed by at least one experienced senior clinician.

### Outcome of therapy

Outcome of therapy was assessed using two different definitions. Survival in respect to the real outcome of therapy was defined as discharge from the hospital. Furthermore, a second analysis was conducted by allocating euthanized calves to the survival group if the calf would likely have lived if unlimited financial resources were available. For this purpose the medical records of euthanized calves were reviewed by three experienced clinicians (FMT, AL, IL) who independently made a decision on the basis of the documented clinical course and the available post-mortem findings. Herewith it was assessed if a calf was more likely to die or to survive if resources for the treatment were unlimited. The final decision was made by a simple majority vote. Results of this analysis are described as the predicted outcome of therapy, compared to the observed outcome of therapy.

### Statistical analyses

Statistical analyses were performed using SPSS for Windows (version 23.0, IBM). GraphPad Prism (version 7.01, GraphPad Software), and the rpart package in R. Values of P < 0.05 were considered to be statistical significant, with P < 0.01 being declared significant for univariate comparisons due to the large dataset and number of comparisons. Data are presented as medians and interquartile ranges (Q_25_/Q_75_) because most of the data were not normally distributed as indicated by the Shapiro-Wilk test and visual examination of QQ-plots. Mann Whitney U-tests were used for comparisons of continuous variables between groups. Also survival rates in relation to deciles of selected laboratory parameters were evaluated. For this purpose a chi-square test was used to compare survival rates of each decile to the survival rate of calves of the decile which best lay within the reference range of the respective laboratory parameter. The level of significance for comparison of nine deciles to the reference decile was adjusted using the Bonferroni-method (*P* ≤ 0.006). Univariate associations between categorized variables and the outcome of therapy was assessed by means of binary logistic regression analysis with calculation of odds ratios (OR) and associated 95% confidence intervals (95% CI).

For multivariate modeling, data was initially analyzed using classification tree analysis in order to identify a pool of potential mortality predictors for subsequent evaluation using logistic regression analysis. Classification tree analysis provides a robust method to recursively partition observed data into an optimal number of subgroups [48], such that calves within each subgroup are similar with respect to a classification variable that is predictive of mortality. Classification trees analyze categorical outcomes; the final result is depicted graphically and resembles an inverted tree with a sequence of branches generated by yes/no questions and answers. As such, classification tree analysis provides an optimized algorithm that can be helpful in clinical decision making. Moreover, the final branched model can be used, as in the study reported here, to identify predictive factors for further statistical analysis. Classification tree analysis handles multicollinearity by identifying the best splitter, makes no assumptions about the distribution of dependent and independent variables, can identify potential outliers and data entry errors, and handles missing data [49].

Classification tree analysis was performed using the rpart package in R [50] and the Gini index to measure node impurity. Calves with missing data were included in the analysis by using surrogate variables. The fitted tree was pruned using the lowest cross-validated error value obtained from 10-fold cross validation; the latter method randomly splits the data set into 10 parts and averages the results for ten classification trees obtained by leaving out one of the 10 parts in turn.

Variables identified as statistically significant predictors during classification tree analysis were subsequently entered as binary outcome variables into multivariate regression models using a stepwise backward procedure with a Wald *P* < 0.01 as selection criterion. Based on the type of available variables, two final models were built: the first model used all potentially useful predictors (clinical and laboratory model), whereas the second model was exclusively based on laboratory findings (laboratory model). The fit of the final logistic regression models was evaluated by means of the Hosmer-Lemeshow Goodness-of-Fit test. The predictive ability of the models were compared by calculating the area under the ROC curve and sensitivity and specificity at the optimal cutpoint identified using the Youden index.

## Results

The medical records of 1,400 calves met the criteria for inclusion in the study, from a total of 9,466 cattle admissions over the same time period (of which 3,277 were calves up to an age of 21 days). The median value and interquartile range for age of calves of the study population was 9.0 (7.0–12.0) d.

### Survival rate

The overall survival rate of calves of this study population was 77.6%. A total of 313 calves (22.4%) did not survive. Of those 313 calves, 61 calves died spontaneously, whereas 252 calves were euthanized on grounds of animal welfare reasons in case of a massive deterioration of the general condition, ongoing depression or anorexia, or in case of severe concurrent disease. In this subset of euthanized calves, an initial treatment trial was performed in 90.1% of cases (n = 227). The grounds for euthanasia were not specifically stated in most of the medical records, but the presumed or obvious reasons are listed in Table 1. In respect to the predicted outcome of therapy a total of 57 euthanized calves were considered as likely survivors after independent reviews of medical records and subsequently allocated to the survival group.

**Table 1:** Assigned reasons for euthanasia of 252 critically ill neonatal calves with diarrhea based on a review of 1,400 medical records.

Reason for euthanasia	Number of calves	Frequency (%)
Ongoing anorexia or insufficient milk intake	58	23.0
Ongoing depression, no improvement in response to treatment	57	22.6
Advanced cachexia and general weakness	45	17.9
Severe neurologic symptoms (e.g. seizures or opisthotonus)	44	17.5
Marked deterioration of general condition	32	12.7
Abdominal emergencies	30	11.9
Complicated navel problems	25	9.9
Septic arthritis	24	9.5
Miscellaneous orthopedic problems (e.g. paresis, phlegmon, gangrene)	24	9.5
Advanced pneumonia	24	9.5
Miscellaneous	18	7.1
Financial constraints in case of multiple concurrent problems	17	6.7
Agonal state	13	5.2
Evidence of ruminitis	9	3.6
Bovine Viral Diarrhea Virus infection	7	2.8

The frequency percentage sums to more than 100% because some calves had more than one reason for euthanasia.

### Post mortem findings

Post-mortem findings were available for 302 out of 313 calves that did not survive to discharge from the hospital. In a total of 262 cases, calves have been submitted to one of two specialized institutes for necropsy. In the remaining 40 calves, post mortem findings were based on intraoperative findings or an in-house necropsy examination which typically did not include an examination of all organ systems. The frequencies of documented findings in the 302 calves are given in Table 2.

**Table 2:** Relative frequencies of documented post-mortem findings in a total of 302 calves with neonatal diarrhea.

Finding	Number of calves^1^	Frequency (%)
Reduced body condition	129/264	48.9
Pneumonia	128/271	47.2
Isolation of *E*. *coli* from multiple organs	123/263	46.8
Markedly reduced body condition/cachexia	71/264	26.9
Lung edema	71/266	26.7
Polyarthritis	67/267	25.1
Extraabdominal navel infections	54/280	19.3
Evidence of septicemia	51/265	19.2
Peritonitis	52/283	18.4
Septic arthritis of one joint	46/268	17.2
Ruminal hyperkeratosis	40/266	15.0
Ileus	39/279	14.0
Meningitis	20/153	13.1
Intraabdominal navel infections	34/278	12.2
Serous atrophy of heart coronary fat	24/264	9.1
Partial lung atelectasis	20/263	7.6
Abomasal ulcers	18/269	6.7
Pleuritis	17/267	6.4
Pericarditis	17/267	6.4
Cerebral edema	8/151	5.3
Ruminitits	12/267	4.5
Malformations	7/265	2.6
Muscle necrosis	7/265	2.6
Bovine Viral Diarrhea Virus infection	7/265	2.6

^1^indicates the proportion of calves where an examination of the respective organ system was documented or expected.

### Univariate associations of study variables with the actual outcome

The number of calves in different clinical categories and the respective proportion of calves with non-survival are given in Table 3. Univariate logistic regression analysis indicated that hypothermia, a depressed or apathetic to comatose behavior, recumbency, absence of the suckling reflex, presence of severe clinical dehydration, and evidence of septicemia were associated with a negative outcome. Presence of concurrent problems that were documented during the first 48 hours of hospitalization such as cachexia, complicated navel infections, bronchopneumonia, neurologic symptoms or abdominal emergencies also resulted in an increased risk for non-survival.

**Table 3:** Results of univariate logistic regression analysis of the association of clinical signs within 48 hours of admission with mortality during hospitalization of 1,400 critically ill neonatal calves with diarrhea.

Variable	Total number	Category (Score)	No. tested	No. (%) with non-survival	OR	95% CI for OR	*P*-Value
**Present on admission**							
Suckling reflex	1,328	Strong (1)	187	19 (10.2)	Ref.		
		Weak (2)	629	97 (15.4)	1.61	0.96–2.72	0.073
		Absent (3)	512	171 (33.4)	**4.43**	**2.67–7.37**	**< 0.001**
Behavior	1,392	bright, alert (1)	517	64 (12.4)	Ref.		
		Depressed (2)	450	86 (19.1)	**1.67**	**1.18–2.38**	**0.004**
		apathetic, comatose (3)	425	160 (37.6)	**4.27**	**3.08–5.93**	**< 0.001**
Posture	1,387	ability to stand (1)	592	84 (14.2)	Ref.		
		impaired ability to stand (2)	383	66 (17.2)	1.26	0.89–1.79	0.20
		Recumbency (3)	412	160 (38.8)	**3.84**	**2.83–5.21**	**< 0.001**
Enophthalmos	1,394	None (1)	436	70 (16.1)	Ref.		
		slight to moderate (2)	664	148 (22.3)	1.50	1.1–2.05	0.011
		Severe (3)	294	92 (31.3)	**2.38**	**1.67–3.40**	**< 0.001**
Hypothermia	1,400	≥ 38.5 (0)	828	142 (17.1)	Ref.		
		< 38.5°C (1)	572	171 (29.9)	**2.06**	**1.60–2.66**	**< 0.001**
SIRS	1,387	No (0)	526	85 (16.2)	Ref.		
		Yes (1)	861	223 (25.9)	**1.81**	**1.37–2.39**	**< 0.001**
**Identified within 48 hours after admission**							
Abdominal emergencies	1,398	No (0)	1,366	283 (20.7)	Ref.		
		Yes (1)	32	28 (87.5)	**26.8**	**9.3–77.0**	**< 0.001**
CNS involvement	1,398	No (0)	1,337	262 (19.6)	Ref.		
		Yes (1)	61	49 (80.3)	**16.8**	**8.8–32.0**	**< 0.001**
Body condition	1,386	Good to moderate (1)	685	103 (15.0)	Ref.		
		Bad (2)	503	110 (21.9)	**1.58**	**1.17–2.13**	**0.003**
		Cachectic (3)	198	98 (49.5)	**5.54**	**3.91–7.85**	**< 0.001**
Orthopedic problems	1,398	No (0)	1,328	271 (20.4)	Ref.		
		Yes (1)	70	40 (57.1)	**5.20**	**3.18–8.50**	**< 0.001**
Predicted septicemia^1^	1,321	No (0)	684	68 (9.9)	Ref.		
		Yes (1)	637	219 (34.4)	**4.75**	**3.52–6.40**	**< 0.001**
Clinical evidence of septicemia	1,387	No (0)	1,165	204 (17.5)	Ref.		
		Yes (1)	222	104 (46.8)	**4.15**	**3.06–5.63**	**< 0.001**
Bronchopneumonia	1,392	No (0)	1,184	216 (18.2)	Ref.		
		Yes (1)	208	90 (43.3)	**3.42**	**2.50–4.67**	**< 0.001**
Navel infections	1,395	None (0)	1,192	238 (20.0)	Ref.		
		Uncomplicated (1)	108	27 (25.0)	1.34	0.85–2.11	0.22
		Complicated (2)	95	43 (45.3)	**3.32**	**2.16–5.09**	**< 0.001**

Ref. = Reference value, SIRS = systemic inflammatory response syndrome.

^1^Presence of septicemia was predicted using a clinical regression model [16].

**Fig 1:**
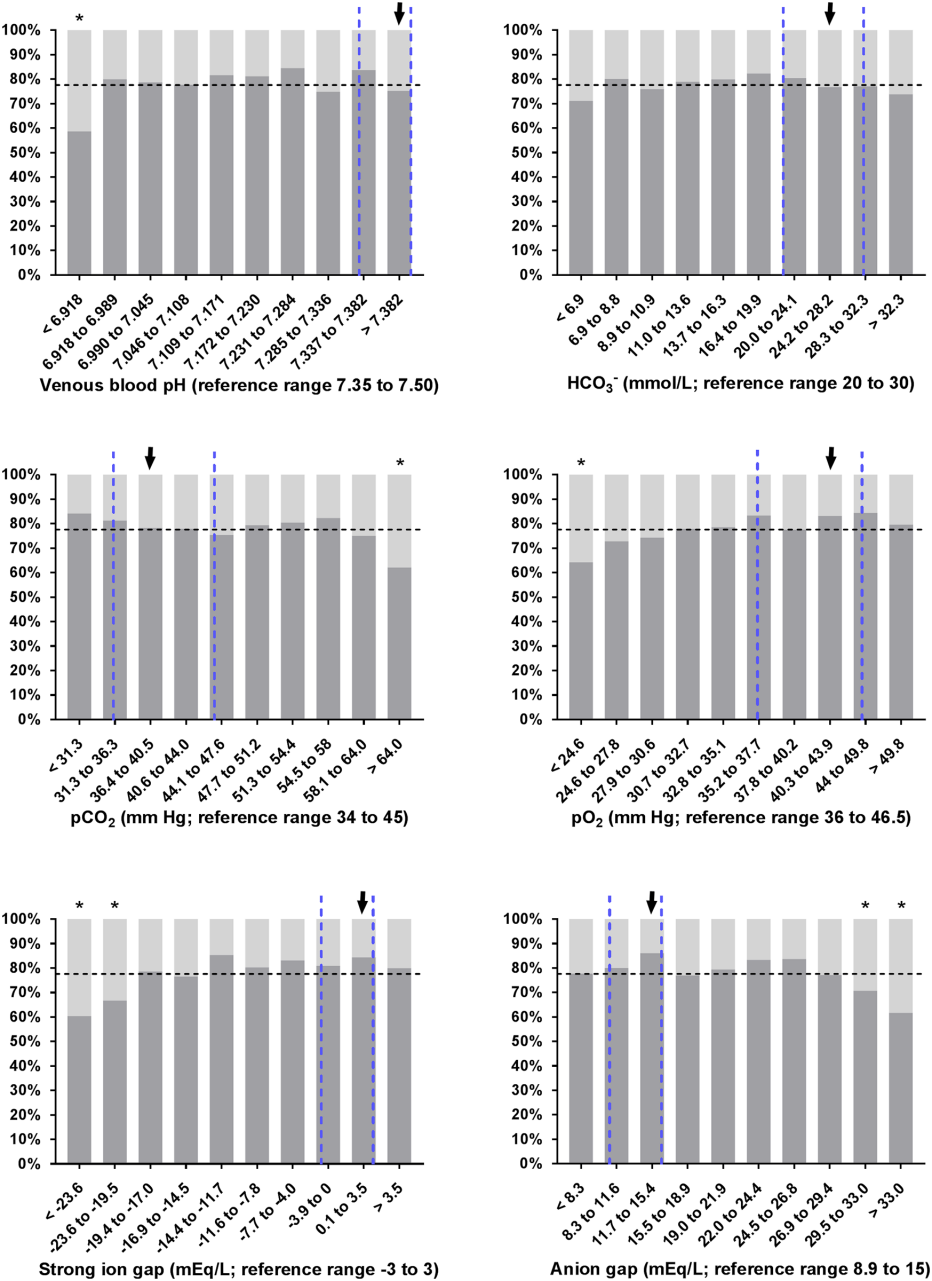
Observed survival rates of 1,400 critically ill neonatal calves with diarrhea in deciles of selected blood gas and acid-base variables. Dashed vertical lines indicate the reference range of respective variables [7, 46, 51] and the dashed horizontal line indicates the overall survival rate of calves of this study population. Survival rates of decile groups that were significantly different (*P* ≤ 0.006) from the survival rate of the reference group (arrow) are indicated by asterisks.

**Fig 2:**
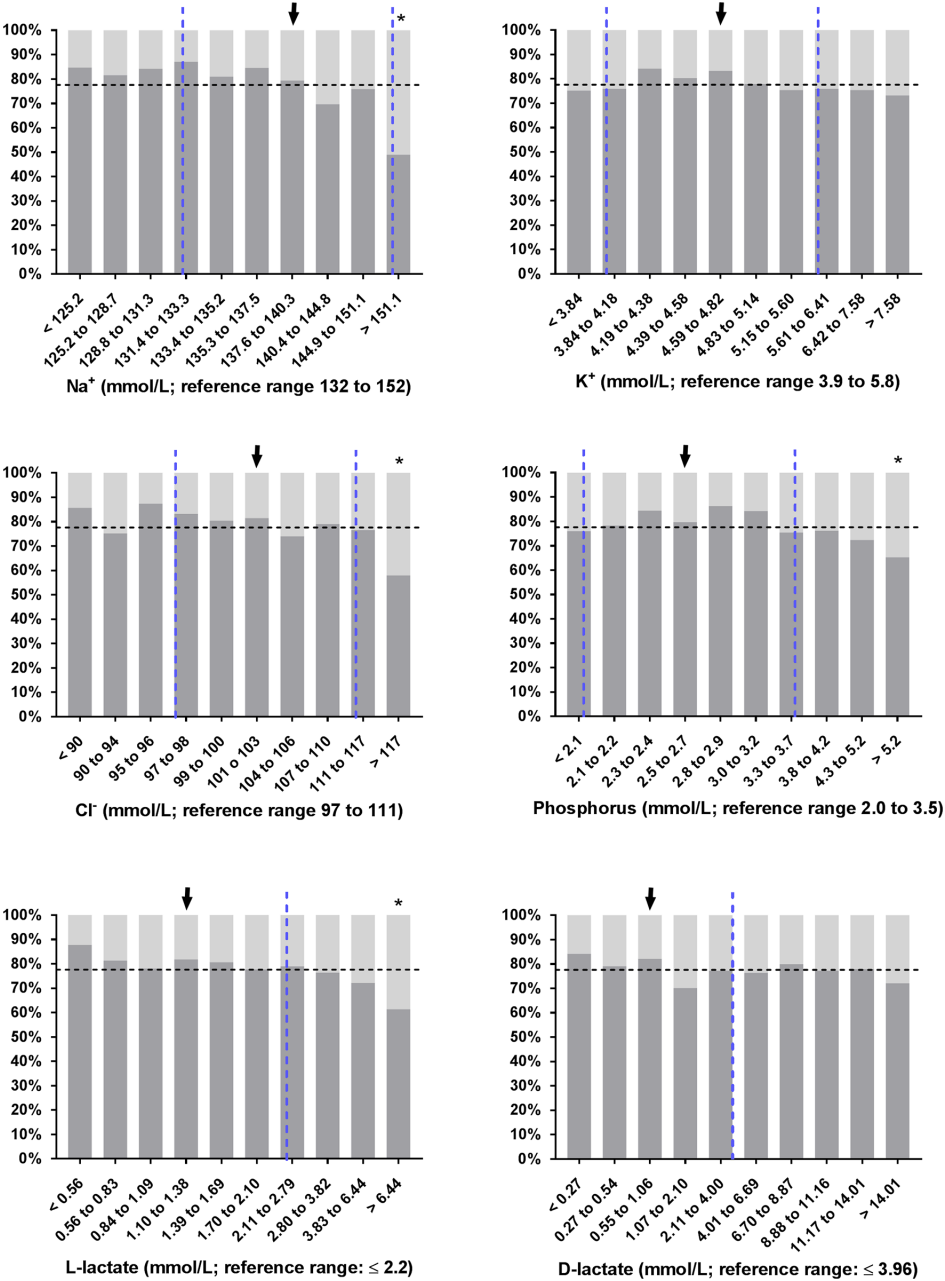
Observed survival rates of 1,400 critically ill neonatal calves with diarrhea in deciles of electrolyte, phosphorus, and L-lactate and D-lactate concentrations. Dashed vertical lines indicate the reference range [40, 46, 51, 52] of respective variables and the dashed horizontal line indicates the overall survival rate of calves of this study population. Survival rates of decile groups that were significantly different (*P* ≤ 0.006) from the survival rate of the reference group (arrow) are indicated by asterisks.

**Fig 3:**
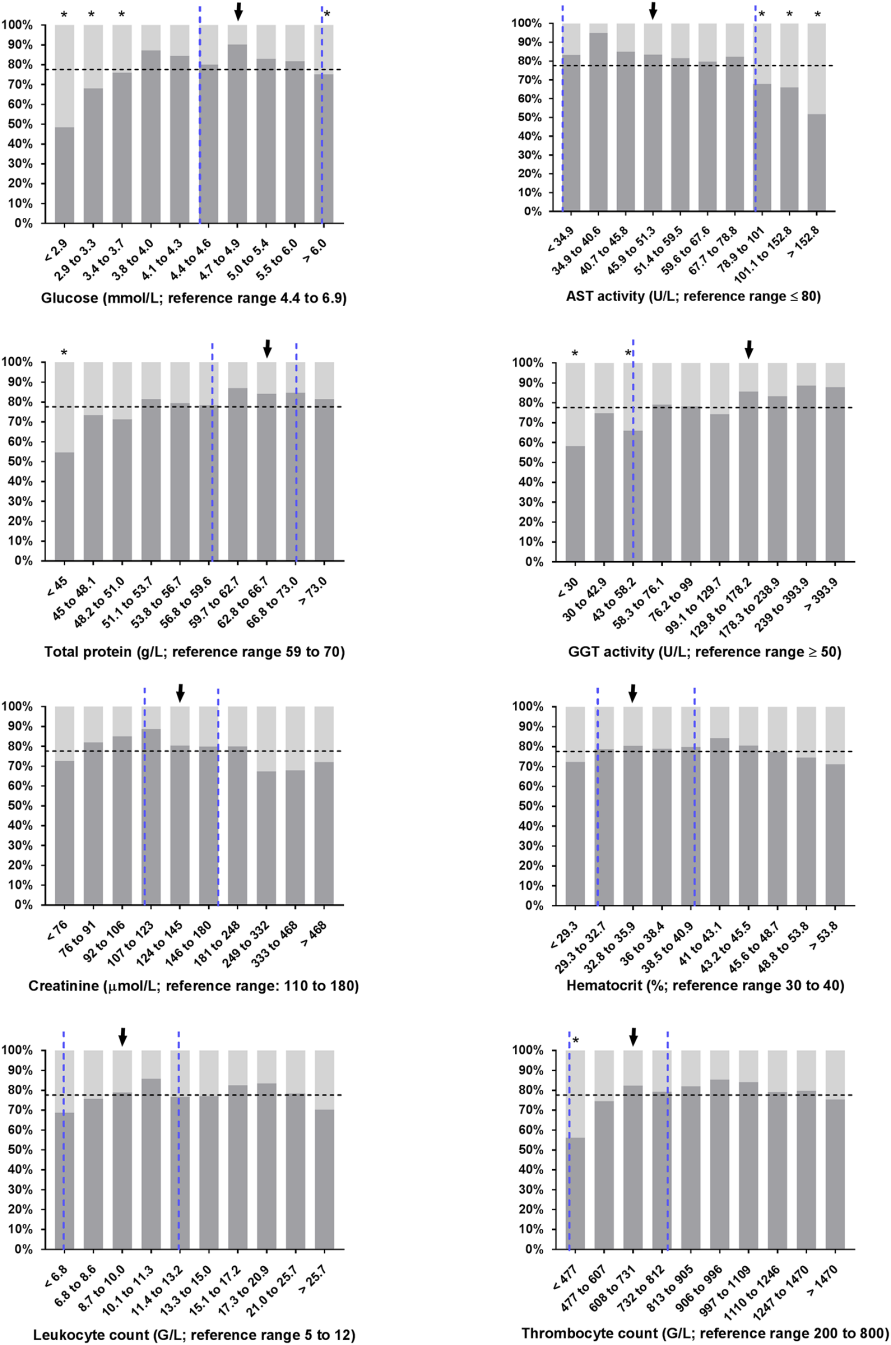
Observed survival rates of 1,400 critically ill neonatal calves with diarrhea in deciles of selected serum/plasma concentrations or activities. Dashed vertical lines indicate the reference range [46, 52] of respective variables and the dashed horizontal line indicates the overall survival rate of calves of this study population. Given that calves in the first two weeks of life with serum gamma glutamyltransferase activity < 50 U/L should be classified as having failure of passive transfer of immunoglobulins [53], this cut-point value was chosen as reference range for this parameter. Survival rates of decile groups that were significantly different (*P* ≤ 0.006) from the survival rate of the reference group (arrow) are indicated by asterisks.

Slight but statistically significant differences between survivors and non-survivors were found for most variables of clinical pathology as shown in Table 4. Interestingly, no difference (*P* > 0.01) was found for base excess, SID_m_, PCV, leukocyte count, and plasma concentrations of bicarbonate, D-Lactate and potassium. The proportion of calves with non-survival in deciles of selected laboratory parameters is shown in Figs 1, 2 and 3. As shown in Fig 2, 51% of calves (n = 73) that were admitted with a sodium concentration > 151.1 mmol/L did not survive. In this subset of calves, 35 out of 59 were euthanized on grounds of dramatic neurologic symptoms, ongoing depression or a massive deterioration of the general condition.

**Table 4:** Laboratory findings on admission of 1,400 critically ill neonatal calves with diarrhea categorized on survival to discharge from the hospital.

Variable	Survivors (n = 1,087) Median (Q_25_/Q_75_)	Non-survivors (n = 313) Median (Q_25_/Q_75_)	*P*-value
***Henderson-Hasselbalch acid-base model***^1^			
Venous blood pH	7.181 (7.030/7.314)	7.133 (6.968/7.308)	**0.005**
pCO_2_ (mm Hg)	47.4 (37.8/55.7)	49.3 (40.5/59.9)	**0.001**
pO_2_ (mm Hg)	35.7 (29.9/42.6)	33.0 (27.1/40.0)	**< 0.001**
HCO_3_^-^ (mmol/L)	16.7 (10.0/25.8)	15.8 (9.6/27.4)	0.84
Base Excess (mmol/L)	-10.8 (-19.8/-0.5)	-12.4 (-21.7/0.3)	0.15
Anion gap (mEq/L)	21.5 (13.3/27.4)	23.5 (15.6/30.8)	**< 0.001**
***Strong ion difference acid-base model***			
A_tot_ (mmol/L)	19.8 (17.5/22.4)	18.2 (16.1/20.8)	**< 0.001**
SID_m_ (mEq/L)	31.5 (24.7/36.8)	31.3 (25.0/38.4)	0.21
USI (mEq/L)	-1.5 (-5.8/3.4)	-2.9 (-9.3/2.0)	**< 0.001**
SIG (mEq/L)	-10.7 (-17.2/-1.1)	-15.0 (-21.6/-4.0)	**< 0.001**
***Electrolytes***			
Na^+^ (mmol/L)	134.7 (129.9/140.4)	139.5 (132.3/150.2)	**< 0.001**
K^+^ (mmol/L)	4.8 (4.3/6.0)	5.0 (4.3/6.3)	0.18
Cl^-^ (mmol/L)	101 (96/107)	104 (98/114)	**< 0.001**
***Clinical biochemical analysis***			
D-lactate (mmol/L)	3.9 (0.7/9.9)	4.6 (1.1/10.3)	0.059
L-Lactate (mmol/L)	1.6 (0.9/3.0)	2.1 (1.2/5.0)	**< 0.001**
Glucose (mmol/L)	4.4 (3.8/5.2)	4.0 (3.0/5.1)	**< 0.001**
Total protein (g/L)	57.7 (50.9/65.4)	53.0 (47.0/60.7)	**< 0.001**
Albumin (g/L)	29.2 (26.7/32.1)	27.9 (25.3/31.1)	**< 0.001**
Globulin (g/L)	28.1 (22.8/34.8)	24.5 (20.0/31.4)	**< 0.001**
Phosphorus (mmol/L)	2.9 (2.4/3.9)	3.3 (2.5/4.4)	**0.001**
Urea (mmol/L)	12.1 (7.2/21.0)	16.8 (10.0/26.9)	**< 0.001**
Creatinine (μmol/L)	140 (99/262)	182 (103/356)	**< 0.001**
**Enzyme activity**^2^			
CK (U/L)	340 (173/831)	653 (261/1964)	**< 0.001**
AST (U/L)	54.8 (41.4/77.5)	80.8 (54.1/133.1)	**< 0.001**
GGT (U/L)	110 (57.5/224.5)	66.9 (36.8/128.6)	**< 0.001**
GLDH (U/L)	7.1 (4.3/13.2)	11.2 (5.5/25.1)	**< 0.001**
**Hematologic analysis**^3^			
PCV (%)	40.9 (34.9/46.7)	41.4 (34.1/48.5)	0.37
Hb (g/dL)	12.8 (11.0/14.6)	12.8 (10.7/15.2)	0.60
MCV (fL)	39.8 (37.6/41.8)	40.7 (38.1/42.9)	**0.001**
MCH (pg)	12.5 (11.7/13.3)	12.5 (11.8/13.3)	0.52
MCHC (g/dL)	31.7 (29.6/33.3)	31.3 (28.9/33.3)	0.031
RDW-CV (%)	21.0 (19.6/23.0)	20.8 (19.2/23.0)	0.18
Leukocytes (G/L)	13.4 (9.6/18.6)	13.2 (8.8/19.9)	0.55
Thrombocytes (G/L)	920 (701/1171)	845 (515/1182)	**0.001**

pCO_2_ = partial pressure of carbon dioxide, pO_2_ = partial pressure of oxygen, A_tot_ = non-volatile weak acids, SID_m_ = measured strong ion difference, USI = unidentified strong ions, SIG = strong ion gap, CK = creatine kinase, AST = aspartate aminotransferase, GGT = gamma glutamyltransferase, GLDH = glutamate dehydrogenase, PCV = packed cell volume, Hb = hemoglobin concentration, MCV = mean corpuscular volume, MCH = mean corpuscular hemoglobin, MCHC = mean corpuscular hemoglobin concentration, RDW = red cell distribution width (coefficient of variation).

^1^Information for pO_2_ was missing in 4 calves with a positive and 1 calf with a negative outcome

^2^Information for AST and GLDH was missing in 2 calves with a positive outcome.

^3^Information was missing in 13 calves with a positive and 7 calves with a negative outcome. Information for RDW-CV was missing in 24 calves with a positive outcome and 12 calves with a negative outcome.

### Multivariate associations between study variables and observed mortality

**Fig 4:**
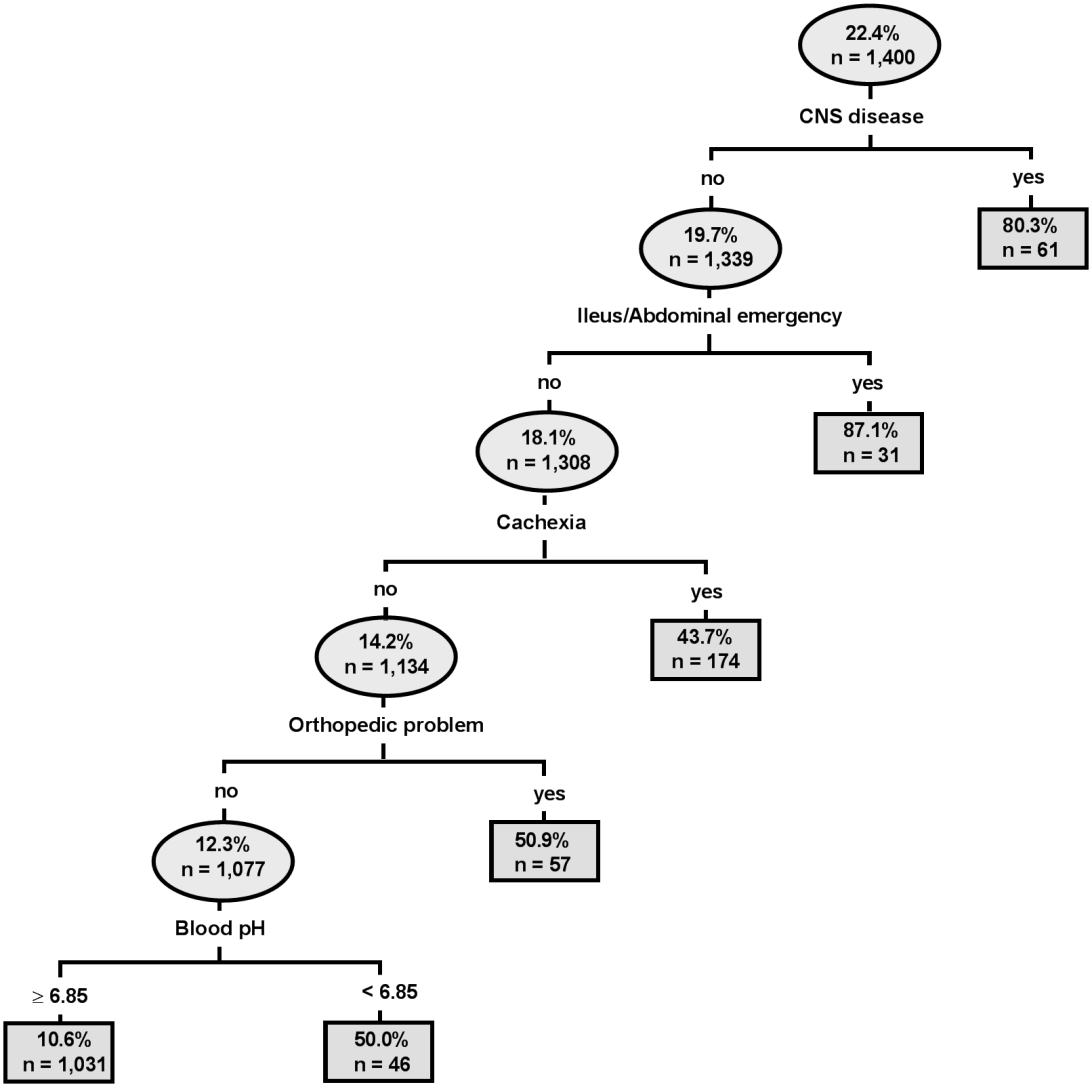
Estimated classification tree identifying significant associations between clinical and laboratory variables and observed mortality. Each oval identifies a subset of the population, the probability of mortality for the subset, and the number of calves in the subset. Lines leaving the oval identify a study variable and its cutpoint value that is a significant predictor of mortality. Branches to the left indicate subgroups with lower mortality (better outcome), whereas branches to the right indicate subgroups with higher mortality (poorer outcome). Classification tree analysis indicated that observed mortality was associated with the presence of central nervous system disease, ileus or abdominal emergency, a cachectic body condition, presence of orthopedic problems, and a jugular venous blood pH < 6.85.

The estimated classification tree for observed calf mortality based on clinical and laboratory data contained 5 branches and is presented in Fig 4. Classification tree analysis indicated that observed calf mortality was associated with four clinical abnormalities (signs of neurologic disease, ileus or abdominal emergency, a cachectic body condition, and presence of orthopedic problems) and only one laboratory abnormality (jugular venous blood pH < 6.85). These 5 variables were then used as independent variables in logistic regression with actual mortality as the outcome (dependent) variable (Table 5).

**Table 5:** Multivariate logistic regression models for identifying associations of clinical and laboratory variables with actual mortality in 1,400 critically ill calves with diarrhea.

Variable	Coefficient	± SE	OR	95% CI for OR	P-value
***Clinical and laboratory model*** ^1^***(n = 1*,*385)***					
Intercept	-2.082	0.098			
Ileus/Abdominal emergency	3.761	0.550	42.98	14.62–126.36	< 0.001
CNS involvement	2.978	0.347	19.65	9.96–38.76	< 0.001
Orthopedic problems	2.037	0.271	7.67	4.51–13.03	< 0.001
Cachectic body condition	1.640	0.181	5.16	3.62–7.35	< 0.001
Venous blood pH < 6.85	1.594	0.292	4.93	2.78–8.73	< 0.001
***Laboratory model***^2^ ***(n = 1*,*380)***					
Intercept	-1.958	0.096			
Sodium concentration ≥ 151 mmol/L	1.462	0.195	4.32	2.95–6.32	< 0.001
Glucose concentration < 3.2 mmol/L	1.282	0.178	3.60	2.54–5.11	< 0.001
GGT activity < 31 U/L	1.075	0.198	2.93	1.99–4.32	< 0.001
Thrombocyte count < 535 G/L	0.846	0.180	2.33	1.64–3.31	< 0.001

Entered predictors were identified by means of Classification Tree analysis.

^1^Hosmer-Lemeshow goodness-of-fit χ^2^ = 2.89, df = 1, *P* = 0.089.

^2^Hosmer-Lemeshow goodness-of-fit χ^2^ = 6.98, df = 3, *P* = 0.073

**Fig 5:**
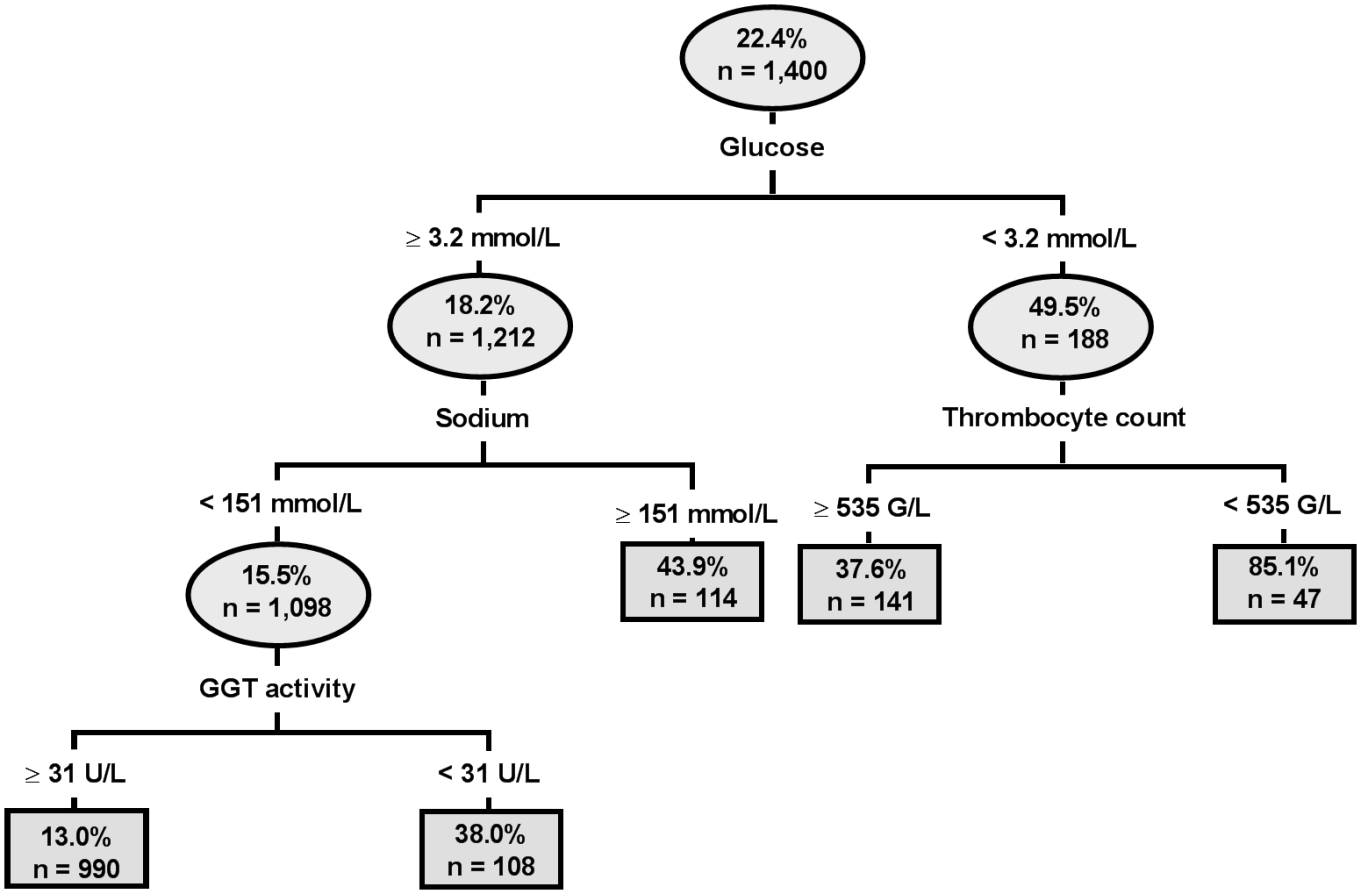
Estimated classification tree identifying significant associations between laboratory variables and observed mortality. Each oval identifies a subset of the population, the probability of mortality for the subset, and the number of calves in the subset. Lines leaving the oval identify a study variable and its cutpoint value that is a significant predictor of mortality. Branches to the left indicate subgroups with lower mortality (better outcome), whereas branches to the right indicate subgroups with higher mortality (poorer outcome). Classification tree analysis indicated that observed mortality was associated with plasma glucose concentrations < 3.2 mmol/L, plasma sodium concentrations ≥ 151 mmol/L, GGT activity < 31 U/L and a thrombocyte count < 535 G/L in hypoglycemic calves.

When exclusively considering laboratory parameters, classification tree analysis identified plasma glucose concentrations < 3.2 mmol/L, plasma sodium concentrations ≥ 151 mmol/L, GGT activity < 31 U/L and a thrombocyte count < 535 G/L in hypoglycemic calves as statistically significant predictors (Fig 5). The results of subsequent logistic regression with those categories of plasma glucose and sodium concentration as independent predictors of actual mortality is also reported in Table 5. The area under the ROC curve (0.77; 95% CI: 0.73–0.80), sensitivity (0.66), and specificity (0.85) of the clinical and laboratory model at the optimal cutpoint (P = 0.25) were higher than that of the laboratory model (area under the ROC curve = 0.71; 95% CI: 0.67–0.75; sensitivity = 0.59; specificity = 0.79; optimal cutpoint, P = 0.27).

When exclusively considering jugular venous blood pH < 6.85, this finding had a sensitivity and specificity of 10.9% and 97.1%, respectively, for predicting non-survival. Of the 1400 calves, 54% (759) had severe acidemia (blood pH < 7.20), 22% (307) had extreme acidemia (blood pH < 7.00), with 35 (3%) had a blood pH < 6.80, with the lowest measured blood pH value being 6.47. The mortality rate was 25% for blood pH < 7.20, 28% for blood pH < 7.00, and 57% for blood pH < 6.80. Fifteen calves survived to discharge with blood pH < 6.80, and the lowest blood pH in a calf that survived was 6.62.

### Univariate associations of study variables with predicted outcome

Univariate logistic regression analysis of clinical and laboratory data indicated that recumbency, an apathetic to comatose behavior, absence of suckling reflex, severe enophthalmos, hypothermia, evidence of SIRS or septicemia, neurologic abnormalities, and abdominal emergencies resulted in a higher risk of non-survival in respect to the predicted than in respect to the actual outcome (S1 Table). In contrast, orthopedic problems, complicated navel infections, or presence of a cachectic body condition resulted in a lower risk of non-survival in terms of predicted than in terms of actual outcome of therapy.

Laboratory data of calves in dependence on the predicted outcome of therapy are provided in S2 Table. Allocation of 57 euthanized calves to the survival group produced minimal changes in the statistical results. Plasma D-lactate concentration and MCHC were the only variables that differed between survivors and non-survivors in respect to the predicted outcome but not in respect to the observed outcome.

### Multivariate associations between study variables and predicted mortality

The clinical and laboratory classification tree for predicted calf mortality contained 11 branches (S1 Fig). Classification tree analysis indicated that predicted calf mortality was associated with the presence of central nervous system disease, ileus or abdominal emergency, abnormal posture (recumbency; score 3), a serum total protein concentration < 43.8 g/L, the presence of pneumonia, a jugular venous pCO_2_ ≥ 65.2 mm Hg in calves with pneumonia, and a packed cell volume ≥ 43.5% in calves with a serum total protein concentration < 43.8 g/L. Also in recumbent calves, serum AST activity ≥ 152 U/L, leukocyte count < 9 G/L, rectal temperature < 35.2°C, and respiratory rate ≥ 62 breaths/min were associated with predicted mortality. When exclusively considering laboratory parameters, classification tree analysis identified serum AST activity > 79 U/L and plasma glucose concentration < 2.9 mmol/L as predictors of mortality (S2 Fig).

These variables were then used as independent variables in logistic regression with predicted mortality as the outcome (dependent) variable. All variables identified as useful predictors during classification tree analysis also reached statistical significance in multivariable regression analysis, except packed cell volume ≥ 43.5% and leukocyte count < 9 G/L which were eliminated at the α = 1% level in the clinical and laboratory model (Table 6). Values for the area under the ROC curve, sensitivity, and specificity were 0.84 (95% CI: 0.81–0.88), 0.69 and 0.88 respectively for the clinical and laboratory model (optimal cutpoint, P = 0.27), and 0.69 (95% CI: 0.65–0.73), 0.62 and 0.72 respectively for the laboratory model (optimal cutpoint, P = 0.19).

**Table 6:** Multivariate logistic regression models for identifying significant associations between study variables and predicted mortality in calves of the present study population. For this analysis euthanized calves were allocated to the survival group if the calf would likely have lived if unlimited financial resources were available. For this purpose medical records were independently reviewed by three experienced clinicians.

Variable	Coefficient	± SE	OR	95% CI for OR	P-value
***Clinical and laboratory model*** ^1^***(n = 1*,*350)***					
Intercept	-3.151	0.158			
Ileus/Abdominal emergency	4.200	0.570	66.67	21.81–203.77	< 0.001
CNS involvement	3.147	0.377	23.27	11.12–48.70	< 0.001
Rectal temperature < 35.2°C	1.736	0.399	5.67	2.60–12.40	< 0.001
Total protein < 43.8 g/L	1.271	0.260	3.56	2.14–5.94	< 0.001
Presence of pneumonia	1.234	0.206	3.44	2.29–5.14	< 0.001
Respiratory rate ≥ 62 breaths/min	1.211	0.315	3.36	1.81–6.23	< 0.001
Recumbency (posture score 3)	1.110	0.187	3.04	2.11–4.37	< 0.001
AST activity ≥ 152 U/L	1.060	0.243	2.89	1.79–4.65	< 0.001
pCO_2_ ≥ 65.2 mm Hg	1.012	0.266	2.75	1.63–4.64	< 0.001
***Laboratory model***^2^ ***(n = 1*,*398)***					
Intercept	-2.161	0.104			
Glucose concentration < 2.9 mmol/L	1.371	0.198	3.94	2.67–5.81	< 0.001
AST activity > 79 U/L	1.227	0.148	3.41	2.55–4.56	< 0.001

Entered predictors were identified by means of classification tree analysis.

^1^Hosmer-Lemeshow goodness-of-fit χ^2^ = 5.55, df = 4, *P* = 0.24.

^2^Hosmer-Lemeshow goodness-of-fit χ^2^ = 0.11, df = 1, *P* = 0.74.

## Discussion

An important finding of this retrospective analysis was that laboratory values in general, and acid-base variables in particular (with the exception being jugular venous blood pH < 6.85), were of limited value for predicting outcome in critically ill neonatal calves with diarrhea. In contrast, the presence of clinical signs reflecting marked neurologic, gastrointestinal, or skeletal/joint dysfunction or profound cachexia were valuable prognostic factors. Our findings strongly suggest that prognosis in critically ill calves with diarrhea can be made more accurate by considering both clinical signs and laboratory values, and support the adage “treat the patient and not the laboratory value”.

Although a statistically significant difference between surviving and non-surviving calves was found for many clinicopathological variables, the statistical significance was primarily due to the large number of calves in the study population. The observed differences in most clinicopathological variables had minimal clinical relevance, as demonstrated by sensitivity estimates of 59% and 62% for actual and predicted outcomes, respectively, determined by multivariable logistic regression. This means that more than 1/3^rd^ of non-surviving calves were not identified by the logistic regression models. More importantly, the logistic regression models that included clinical signs and laboratory values had a higher predictive ability than logistic regression models based on clinicopathological data alone.

Severe acidemia due to metabolic acidosis in critically ill humans is usually defined as blood pH < 7.20 and is associated with poorer clinical outcomes and a higher mortality rate [1, 4, 5, 54]. Severe acidemia has also been associated with serious clinical and metabolic manifestations such as decreased cardiac contractility, cardiac output, and glomerular filtration rate, gastric atony, decreased hepatic blood flow, insulin resistance, and pulmonary edema, thereby complicating the clinical management of affected patients [55, 56]. Since such patients also usually suffer from sepsis, hepatic and renal failure, intoxications or hypoxemic conditions it remains controversial whether severe acidemia is an etiologic contributor to organ dysfunction or just a marker of systemic illness and hyper-L-lactatemia [1]. However, acid-base homeostasis exerts a major influence on protein function and therefore critically affects tissue and organ performance [55] which is supported by experimental studies in piglets and dogs indicating that severe acidemia might be at least a contributor to organ dysfunction [57, 58]. The study population used for the study reported here was unique in that 307 calves had extreme acidemia, defined as a jugular venous blood pH < 7.00 [59], and 35 of those calves had a blood pH < 6.80. To our knowledge, this study population represents the largest number of critically ill patients studied with extreme acidemia [59, 60]. As a result, this study population provided an opportunity to determine the effects of extreme acidemia on survival in critically ill calves with naturally occurring disease. A remarkable finding of the classification tree analysis was that jugular venous blood pH was predictive of non-survival, but only when blood pH < 6.85. Moreover, plasma bicarbonate concentrations and base excess were similar for survivors and non-survivors, and values for SIG (representing the unmeasured strong ion concentration and thereby a major contributor to strong ion acidosis and acidemia [9]) were not predictive for mortality in the multivariate analysis. Other findings of interest were that the non-survival rate for calves with blood pH < 7.00 was 28%, and 57% for blood pH < 6.80. Mortality rates in critically ill humans with blood pH < 7.00 vary from 90% for patients with cardiac arrest before admission, 66% for septic shock, and 22% for cases linked with diabetes mellitus [59]. Capillary blood pH can decrease to 6.80 in elite track and field athletes undergoing a short exercise program [61] and venous blood pH decreased to 6.74 in an Olympic gold medalist oarsman undergoing a simulated race on a rowing ergometer [62]. These acute decreases in blood pH appear to be well tolerated in healthy subjects. Taken together, these findings suggest that the nature and severity of the underlying clinical condition, rather than the blood pH value per se, has a more important effect on survival.

A significant difference for plasma D-lactate concentrations between calves with a positive and negative outcome was only identified in respect to the predicted outcome, but was not identified as a significant predictor during classification tree analysis. These findings clearly indicate that neonatal diarrheic calves can recover from severe acidemia and D-lactatemia if treated properly. The same conclusion was also made in a previous study where high D-lactate concentrations had no impact on the outcome of 300 hospitalized neonatal diarrheic calves [8]. Intravenous administration of sodium bicarbonate solutions are the treatment of choice in diarrheic calves with clinical signs of (D-lactic) acidosis, and studies in diarrheic calves [63, 64], and neonatal kids [19] have indicated that markedly elevated plasma D-lactate concentrations normalize after correction of metabolic acidosis. The reasons for this phenomenon are yet not fully understood but appear to be related to an increased glomerular filtration rate after rehydration and correction of systemic acidosis per se, which might have an effect on gastrointestinal production and/or metabolism of D-lactate [65].

Both in human and veterinary medicine, L-lactate is a well-established biomarker of tissue hypoxia, sepsis, disease severity and mortality [4, 66]. Measurement of blood L-lactate concentration has also attracted increasing attention in bovine medicine due to the widespread availability and validation of inexpensive and portable L-lactate analyzers [67]. In critically ill neonatal foals admitted to intensive care units with common problems such as bacterial infections, perinatal asphyxia, prematurity or enteritis, increased blood or plasma L-lactate concentrations are associated with bacteremia and evidence of SIRS and are considered to provide clinically useful indicators of non-survival [22, 23]. Although high L-lactate concentrations were associated with an increased risk of mortality in the present study (Fig 2), hyper L-lactatemia was not identified as a useful predictor during classification tree analysis. This could be explained by the majority of calves of the study population having normal or slightly elevated L-lactate concentrations (Fig 2) and by underlying conditions, separate to severe dehydration, that may result in hyper L-lactatemia in critically ill calves with diarrhea. However, prognostic studies in septic children and adult humans also found a diagnostic overlap between surviving and non-surviving patients and reported that serial L-Lactate measurements for monitoring the subsequent decrease of L-lactate in response to therapy provided more reliable information than a single measurement of L-lactate on admission [68, 69]. Similar findings have been reported for critically ill foals and adult equine emergencies where a delayed normalization or persistent L-lactatemia represented a more reliable indicator of non-survival than admission L-lactate concentrations [23, 70, 71]. Further studies appear indicated to assess whether serial measurements of blood or plasma L-lactate concentrations in critically ill calves provides more reliable prognostic information than a single measurement before the initiation of therapy.

Amongst the laboratory parameters a plasma sodium concentration ≥ 151 mmol/L was one of the few statistically significant predictors that could be identified during classification tree analysis. This finding was consistent with calves in the 10^th^ decile of plasma sodium concentrations (*c*Na^+^ > 151.1 mmol/L) having an observed mortality rate of 51% (Fig 2). In general, hypernatremia can result from excessive free water loss resulting in hypertonic dehydration (which is unusual in diarrheic calves), administration of oral or parenteral fluids with a high sodium content to animals with limited or no access to free water, ingestion of sodium-containing salts without ingesting an adequate volume of water, or combinations of these factors [72]. Cases of hypernatremia and salt intoxications in neonatal diarrheic calves have also been reported previously and were mostly attributed to mixing errors of oral electrolyte solutions [73–75]. In a retrospective analysis involving 163 neonatal hospitalized calves with hypernatremia (including some of the cases in the present study population), this condition was significantly associated with administration of oral electrolyte solutions, but also to pretreatment with (hypertonic) sodium bicarbonate containing infusion solutions and no access to free water prior to hospitalization [76].

The consequences of hypernatremia depend on the magnitude and duration of this electrolyte disorder. Experimental in vitro and in vivo studies in rats and rabbits have shown that marked acute hypernatremia can result in central nervous system dysfunction due to cellular dehydration, myelinolysis, cellular necrosis and inhibition of neuronal cell glycolysis [77, 78]. During chronic hypernatremia, osmoregulation in the brain occurs by intracellular accumulation of organic osmoles such as amino acids, sugars, and alcohols in an attempt to counterbalance the differences between intra- and extracellular osmolality [77, 79]. The latter homeostatic response complicates the clinical management of hypernatremic animals, as it predisposes to the development of cerebral edema by intracellular fluid shifts if intravenous fluid therapy is necessary as it was in most of the calves in the study reported here. Early clinical signs of hypernatremia in calves are severe depression and lethargy [73], and tremors, seizures, opisthotonus and coma in more advanced stages, causing a clinical picture which is usually called salt poisoning or water intoxication [72, 80]. In the present study the large majority of hypernatremic calves that did not survive to discharge were euthanized for reasons of a massive deterioration of the general condition, ongoing depression or dramatic neurological symptoms indicating that hypernatremia was indeed a direct cause for a negative outcome.

Another condition which was significantly associated with non-survival was hypoglycemia. Although hypoglycemia has been reported in diarrheic calves [81, 82], marked hypoglycemia had a low prevalence in the present study population as calves in the first decile of plasma glucose concentrations had values up to 2.8 mmol/L. Although marked hypoglycemia may be due, in part, to a prolonged period since the last milk feeding or administration of low glucose content oral electrolyte solutions before admission [83], calves in the first decile of plasma glucose concentration had an observed lethality rate of 51.5%. Similar findings were also reported in a recent retrospective analysis involving data from 10,060 neonatal hospitalized calves [24]. In that study, severe hypoglycemia (plasma glucose concentration < 2.0 mmol/L) was present in only 6.3% of cases but resulted in a mortality rate of 79.4%. The high fatality rate in markedly hypoglycemic calves can be explained by serious health conditions associated with this metabolic imbalance, including malnutrition, abdominal emergencies including generalized peritonitis of varied origin, and clinical or post-mortem evidence of septicemia [24]. The association between hypoglycemia and septicemia/endotoxemia in neonatal calves has been demonstrated in experimental studies after intravenous *E*. *coli* or endotoxin challenges [84, 85], indicating that hypoglycemia in diarrheic calves should alert the clinician to the potential presence of septicemia. This supposition is supported by the results of a multicenter retrospective analysis of blood glucose concentrations in 515 critically ill neonatal foals, where marked hypoglycemia was associated with a poor outcome and a diagnosis of sepsis or positive bacteriological blood culture [25].

Review of the necropsy reports of calves with a negative outcome additionally indicated that a large proportion of calves suffered from septicemia or resulting consequences such as septic arthritis or septic inflammation of other body cavities (Table 2). In a study conducted on a calf rearing farm, bacteremia was found in 28% of calves with signs of diarrhea or depression [86]. Similarly, septicemia was diagnosed in 31% of 252 hospitalized diarrheic calves [16], strongly indicating that those conditions are common complications in neonatal calf diarrhea. In both studies, failure of transfer of passive immunity was considered a major risk factor for the presence of bacteremia and septicemia, and was associated with a poor outcome. Failure of transfer of passive immunity is also associated with an increased duration of diarrhea in neonatal calves [87]. Unfortunately, plasma IgG concentrations were not measured in calves of the present study population but we identified a low serum GGT activity < 31 U/L as a risk factor for non-survival (Fig 5). Cows have a high GGT activity in colostrum and the serum activities of GGT in calves that have suckled and ingested a sufficient amount of colostrum can be 60 to 160 times greater than in adult animals [88, 89], such that GGT activity can be used as an indirect age-dependent measure of passive transfer of immunoglobulins [53]. Calves in the first 2 weeks of age with a serum GGT activity < 50 U/L should be classified as having failure of transfer of passive immunity [53]. Failure of immunoglobulin transfer represents also an explanation for the association between low total protein concentrations and observed mortality (Figs 3 and S1). An association between low serum total protein concentration and an increased risk of mortality during the first weeks of live has also been demonstrated previously [90] and in a recent study a serum total protein concentration of 58 to 63 g/L has been recommended as an endpoint to indicate adequate transfer of passive immunity based on observed mortality events during the first 4 months of life [91]. However, in the present study it needs to be considered that this parameter was measured on admission to the hospital and was therefore also influenced by varying degrees of dehydration. Consequently we would have expected a higher predictive ability if total protein concentration had been measured after rehydration of calves. In the study by Lofstedt et al. [16], the presence of focal infections was also identified as a major risk factor for septicemia. This could also be an explanation for the prognostic relevance of pneumonia or complicated navel infection in the present study; however, those findings were also a direct reason for euthanasia.

In addition to signs of central nervous system involvement, ileus or abdominal emergencies, or orthopedic problems, a poor body condition and especially cachexia was identified as a risk factor for the observed negative outcome in this study population (Fig 4). The latter condition was likely related to feeding of inadequately low volumes of milk, milk withdrawal, low intake of offered milk volumes, prolonged duration of illness, or combinations of those before admission to the hospital. Continued feeding of milk was traditionally thought to aggravate diarrhea in calves and withdrawal of milk is therefore still performed by farmers, although there is no scientific evidence to support the practice. Malnutrition resulting in emaciation and a clinical picture characterized by general weakness, impaired ability to stand or inability to suckle was a direct reason for euthanasia in calves of the present study. Severe emaciation might also have negatively impacted the immune response of affected calves and therefore favor any concurrent problems resulting in a negative outcome. Our findings emphasize the importance of providing a sufficient energy supply to neonatal calves with diarrhea.

Although this study provided valuable information in respect to prognostic factors in neonatal calf diarrhea, our study has several limitations. First of all, our analysis was based on a university hospital population where the caseload is usually preselected towards more severely affected cases. Furthermore, not all calves were admitted to the hospital as first opinion cases and any pretreatment activities might therefore have affected the outcome of our analysis. Also, this study was retrospective which has potential limitations due to lack of standardized documentation and categorization of clinical findings, and individual treatment variations which were not considered in the present analysis. An obvious strength of our study is, however, that blood samples of critically ill calves were collected and analyzed in the same manner and that acid-base variables were measured using the same blood gas analyzer. Consequently, our data provided a unique opportunity to study the prognostic relevance of acid-base variables and specific findings of clinical biochemistry in a large study population.

Another factor which might have affected the outcome of our analysis is the fact that high proportions (81%) of calves categorized as non-survivors were euthanized. Although euthanasia appeared to be justified for medical or animal welfare reasons and was supported by the post-mortem findings in the majority of cases (Table 2), the decision to euthanize a calf did not follow standardized criteria. Consequently, it cannot be completely ruled out that some calves would have recovered with intensive and high-cost therapy (e.g. calves with a diagnosis of septic arthritis). This issue was addressed by an independent review of the medical records of euthanized calves and creation of a second outcome definition. However, those additional analyses did not change any of the major conclusions.

We believe that some of the findings in the study reported here are translatable to the treatment of diarrheic children under five years of age. An interesting comparative aspect of our study was that classification tree analysis identified four clinical abnormalities (neurologic disease, abdominal emergencies including ileus, cachexia, and orthopedic disease which is primarily septic arthritis) that were independent predictors of mortality in diarrheic calves. The first three clinical abnormalities are predictive of mortality in diarrheic children under five years of age, with neurologic disease manifest as drowsiness, abnormal mentation and convulsions, abdominal abnormalities manifest as ileus and abdominal distention, and cachexia manifest as severe malnutrition [28, 32–34]. A prominent difference between diarrheic calves and children is the rarity of septic arthritis in diarrheic children [92], whereas septic arthritis of one or more joints was identified in 8.1% (113/1400) of the diarrheic calves in this study. We cannot identify a reason for this marked difference in the prevalence of septic arthritis. Another interesting comparative observation is that studies in diarrheic children that evaluated both clinical and laboratory predictors of mortality always identified at least one clinical abnormality as being predictive [28, 32, 93]. In other words, evaluating the predictive ability of both laboratory and clinical findings consistently resulted in logistic regression models of greater explanatory ability in predicting death in diarrheic children than models based on laboratory values alone.

## Conclusions

The findings of the study reported here clearly indicate that laboratory parameters are not as valuable as clinical findings when predicting the response of critically ill hospitalized neonatal diarrheic calves to treatment. We therefore conclude that it can be worthwhile to treat calves with severe metabolic derangements as long as clinical signs of neurologic, abdominal, or orthopedic disease with a more guarded prognosis are not evident.

## Supporting information

S1 Fig Estimated classification tree identifying significant associations between clinical and laboratory variables and predicted mortality. Each oval identifies a subset of the population, the probability of mortality for the subset, and the number of calves in the subset. Lines leaving the oval identify a study variable and its cutpoint value that is a significant predictor of mortality. Branches to the left indicate subgroups with lower mortality (better outcome), whereas branches to the right indicate subgroups with higher mortality (poorer outcome). Classification tree analysis suggests that predicted mortality is associated with the presence of central nervous system disease, ileus or abdominal emergency, abnormal posture, a serum total protein concentration < 43.8 g/L, the presence of pneumonia, a jugular venous pCO_2_ ≥ 65.2 mm Hg in calves with pneumonia, and a packed cell volume ≥ 43.5% in calves with a serum total protein concentration < 43.8 g/L. Also in recumbent calves, a serum AST activity ≥ 152 U/L, a leukocyte count < 9 G/L, a rectal temperature < 35.2°C and a respiratory rate ≥ 62 breaths/min was significantly associated with predicted mortality.
(PDF)

S2 Fig Estimated classification tree identifying significant associations between laboratory variables and predicted mortality. Each oval identifies a subset of the population, the probability of mortality for the subset, and the number of calves in the subset. Lines leaving the oval identify a study variable and its cutpoint value that is a significant predictor of mortality. Branches to the left indicate subgroups with lower mortality (better outcome), whereas branches to the right indicate subgroups with higher mortality (poorer outcome). Classification tree analysis suggests that predicted mortality is associated with a serum AST activity > 79 U/L and plasma glucose concentrations < 2.9 mmol/L.
(PDF)

S1 Table Results of a univariate logistic regression analysis for identifying clinical variables that were associated with predicted mortality in 1,400 critically ill neonatal calves with diarrhea. (DOCX)

S2 Table Laboratory findings in 1,400 critically ill neonatal calves with diarrhea in dependence on the predicted outcome of therapy. (DOCX)
