# Supplementary material for: Clinical signs, profound acidemia, hypoglycemia, and hypernatremia are predictive of mortality in 1,400 critically ill neonatal calves with diarrhea
Source: PLoS One. 2017 Aug 17;12(8):e0182938. doi: 10.1371/journal.pone.0182938 (PMC5560544; doi:10.1371/journal.pone.0182938)
Supplement: S2 Fig — Each oval identifies a subset of the population, the probability of mortality for the subset, and the number of calves in the subset. Lines leaving the oval identify a study variable and its cutpoint value that is a significant predictor of mortality. Branches to the left indicate subgroups with lower mortality (better outcome), whereas branches to the right indicate subgroups with higher mortality (poorer outcome). Classification tree analysis suggests that predicted mortality is associated with a serum AST activity > 79 U/L and plasma glucose concentrations < 2.9 mmol/L. (PDF) [file pone.0182938.s002.pdf]

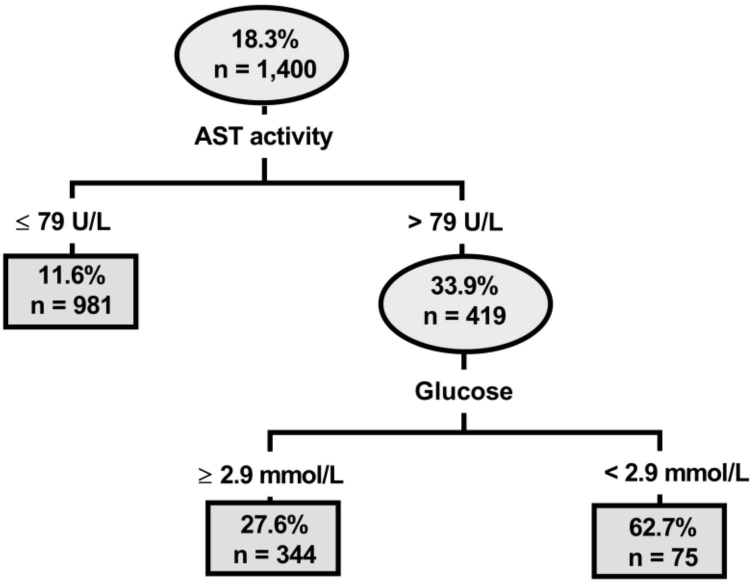

S2 Figure. Estimated classification tree identifying significant associations between laboratory variables and predicted mortality. Each oval identifies a subset of the population, the probability of mortality for the subset, and the number of calves in the subset. Lines leaving the oval identify a study variable and its cutpoint value that is a significant predictor of mortality. Branches to the left indicate subgroups with lower mortality (better outcome), whereas branches to the right indicate subgroups with higher mortality (poorer outcome). Classification tree analysis suggests that predicted mortality is associated with a serum AST activity > 79 U/L and plasma glucose concentrations < 2.9 mmol/L.
