# Supplementary material for: Clinical signs, profound acidemia, hypoglycemia, and hypernatremia are predictive of mortality in 1,400 critically ill neonatal calves with diarrhea
Source: PLoS One. 2017 Aug 17;12(8):e0182938. doi: 10.1371/journal.pone.0182938 (PMC5560544; doi:10.1371/journal.pone.0182938)
Supplement: S1 Table — (DOCX) [file pone.0182938.s003.docx]

**S1 Table. Results of a univariate logistic regression analysis for identifying clinical variables that were associated with predicted mortality in 1,400 critically ill neonatal calves with diarrhea.**

| **Variable** | **Total number** | **Category (Score)** | **No.**  **tested** | **No. (%) with non-survival** | **OR** | **95% CI for OR** | ***P*-Value** |
| --- | --- | --- | --- | --- | --- | --- | --- |
| **Present on admission** | | |  |  |  |  |  |
| Suckling reflex | 1,328 | Strong (1) | 187 | 12 (6.4) | Ref. |  |  |
|  |  | Weak (2) | 629 | 71 (11.3) | 1.86 | 0.98-3.50 | 0.056 |
|  |  | Absent (3) | 512 | 152 (29.7) | **6.16** | **3.33-11.39** | **< 0.001** |
| Behavior | 1,392 | bright, alert (1) | 517 | 41 (7.9) | Ref. |  |  |
|  |  | Depressed (2) | 450 | 68 (15.1) | **2.07** | **1.37-3.12** | **0.001** |
|  |  | apathetic, comatose (3) | 425 | 144 (33.9) | **5.95** | **4.08-8.67** | **< 0.001** |
| Posture | 1,387 | ability to stand (1) | 592 | 58 (9.8) | Ref. |  |  |
|  |  | impaired ability to  stand (2) | 383 | 52 (13.6) | 1.45 | 0.97-2.16 | 0.070 |
|  |  | Recumbency (3) | 412 | 143 (34.7) | **4.89** | **3.49-6.87** | **< 0.001** |
| Enophthalmos | 1,394 | None (1) | 436 | 51 (11.7) |  |  |  |
|  |  | slight to moderate (2) | 664 | 123 (18.5) | **1.72** | **1.21-2.44** | **0.003** |
|  |  | Severe (3) | 294 | 79 (26.9) | **2.77** | **1.88-4.10** | **< 0.001** |
| Hypothermia | 1,400 | ≥ 38.5 (0) | 828 | 108 (13.0) |  |  |  |
|  |  | < 38.5° C (1) | 572 | 148 (25.9) | **2.33** | **1.77-3.07** | **< 0.001** |
| SIRS | 1,387 | No (0) | 526 | 61 (11.6) |  |  |  |
|  |  | Yes (1) | 861 | 191 (22.2) | **2.17** | **1.59-2.97** | **< 0.001** |
| **Identified within 48 hours after admission** | | |  |  |  |  |  |
| Abdominal  emergencies | 1,398 | No (0) | 1,366 | 226 (16.5) | Ref. |  |  |
|  |  | Yes (1) | 32 | 28 (87.5) | **35.3** | **12.3-101.6** | **< 0.001** |
| CNS involvement | 1,398 | No (0) | 1,337 | 205 (15.3) | Ref. |  |  |
|  |  | Yes (1) | 61 | 49 (80.3) | **22.5** | **11.8-43.1** | **< 0.001** |
| Predicted septicemia^1^ | 1,321 | No (0) | 684 | 49 (7.2) | Ref. |  |  |
|  |  | Yes(1) | 637 | 186 (29.2) | **5.35** | **3.82-7.49** | **< 0.001** |
| Body condition | 1,386 | Good to moderate (1) | 685 | 86 (12.6) | Ref. |  |  |
|  |  | Bad (2) | 503 | 88 (17.5) | 1.48 | 1.07-2.04 | 0.018 |
|  |  | Cachectic (3) | 198 | 80 (40.4) | **4.72** | **3.29-6.79** | **< 0.001** |
| Clinical evidence of  septicemia | 1.387 | No (0) | 1,165 | 161 (13.8) | Ref. |  |  |
|  |  | Yes (1) | 222 | 91 (41) | **4.33** | **3.16-5.94** | **< 0.001** |
| Bronchopneumonia | 1,392 | No (0) | 1,184 | 171 (14.4) | Ref. |  |  |
|  |  | Yes (1) | 208 | 79 (38.0) | **3.63** | **2.63-5.01** | **< 0.001** |
| Navel infections | 1,395 | None (0) | 1,192 | 197 (16.5) | Ref. |  |  |
|  |  | Uncomplicated (1) | 108 | 24 (22.2) | 1.44 | 0.89-2.33 | 0.13 |
|  |  | Complicated (2) | 95 | 30 (31.6) | **2.33** | **1.47-3.69** | **< 0.001** |
| Orthopedic problems | 1,398 | No (0) | 1,328 | 230 (17.3) | Ref. |  |  |
|  |  | Yes (1) | 70 | 24 (34.3) | **2.49** | **1.49-4.16** | **< 0.001** |

Ref. = Reference value, ^1^Presence of septicemia was predicted using a clinical regression model [16]. SIRS = systemic inflammatory response syndrome
