# Supplementary material for: Clinical signs, profound acidemia, hypoglycemia, and hypernatremia are predictive of mortality in 1,400 critically ill neonatal calves with diarrhea
Source: PLoS One. 2017 Aug 17;12(8):e0182938. doi: 10.1371/journal.pone.0182938 (PMC5560544; doi:10.1371/journal.pone.0182938)
Supplement: S2 Table — (DOCX) [file pone.0182938.s004.docx]

**S2 Table. Laboratory findings in 1,400 critically ill neonatal calves with diarrhea in dependence on the predicted outcome of therapy.**

| **Variable** | **Survivors**  **(n = 1,144)**  **Median (Q_25_/Q_75_)** | **Non-survivors**  **(n = 256)**  **Median (Q_25_/Q_75_)** | ***P*-value** |
| --- | --- | --- | --- |
| ***Henderson-Hasselbalch acid-base model^1^*** | |  |  |
| Venous blood pH | 7.182 (7.030/7.318) | 7.113 (6.951/7.292) | **< 0.001** |
| pCO_2_ (mm Hg) | 47.6 (38.0/55.9) | 48.6 (40.1/60.6) | 0.013 |
| pO_2_ (mm Hg) | 35.7 (29.9/42.3) | 33.0 (27.0/40.1) | **< 0.001** |
| HCO_3_^-^ (mmol/L) | 16.8 (10.1/25.9) | 15.0 (9.3/27.2) | 0.20 |
| Base Excess (mmol/L) | -10.8 (-19.8/-0.1) | -14.3 (-22.4/-0.3) | 0.012 |
| Anion gap (mEq/L) | 21.2 (13.1/27.4) | 25.0 (17.2/31.1) | **< 0.001** |
| ***Strong ion difference acid-base model*** | |  |  |
| A_tot_ (mmol/L) | 19.8 (17.3/22.4) | 18.1 (16.1/20.8) | < **0.001** |
| SID_m_ (mEq/L) | 31.6 (24.8/37) | 30.8 (24.7/38.3) | 0.53 |
| USI (mEq/L) | -1.4 (-5.9/3.5) | -3.8 (-9.4/1.3) | **< 0.001** |
| SIG (mEq/L) | -10.5 (-17.3/-1.0) | -15.6 (-22.1/-6.6) | **< 0.001** |
| ***Electrolytes*** |  |  |  |
| Na^+^ (mmol/L) | 134.7 (130.0/140.4) | 141.3 (133.2/151.4) | **< 0.001** |
| K^+^ (mmol/L) | 4.8 (4.3/6.0) | 5.0 (4.3/6.4) | 0.12 |
| Cl^-^ (mmol/L) | 101 (96/107) | 104 (98/116) | **< 0.001** |
| ***Clinical biochemistry analysis*** | |  |  |
| D-lactate (mmol/L) | 3.8 (0.7/9.8) | 5.3 (1.4/10.7) | **0.005** |
| L-Lactate (mmol/L) | 1.6 (0.9/3.0) | 2.1 (1.2/5.2) | **< 0.001** |
| Glucose (mmol/L) | 4.4 (3.8/5.2) | 3.8 (2.8/5.0) | **< 0.001** |
| Total protein (g/L) | 57.7 (50.5/65.2) | 52.8 (46.9/60.5) | **< 0.001** |
| Albumin (g/L) | 29.2 (26.6/32.1) | 27.7 (25.2/31.1) | **< 0.001** |
| Globulin (g/L) | 28.0 (22.6/34.7) | 24.6 (20.0/31.4) | **< 0.001** |
| Phosphorus (mmol/L) | 2.9 (2.4/3.9) | 3.4 (2.5/4.5) | **< 0.001** |
| Urea (mmol/L) | 12.2 (7.1/21.0) | 17.6 (11.0/27.8) | **< 0.001** |
| Creatinine (µmol/L) | 139 (98/262) | 194 (118/371) | **< 0.001** |
| **Enzyme activity^2^** |  |  |  |
| CK (U/L) | 339 (170/832) | 813 (309/2154) | **< 0.001** |
| AST (U/L) | 55.1 (41.4/77.7) | 87.1 (57.8/151.5) | **< 0.001** |
| GGT (U/L) | 108.4 (54.7/221) | 67.6 (38.0/128.8) | **< 0.001** |
| GLDH (U/L) | 7.1 (4.2/13.3) | 11.8 (5.8/31.1) | **< 0.001** |
| **Hematologic analysis^3^** | |  |  |
| PCV (%) | 40.9 (34.4/46.8) | 41.7 (34.6/48.3) | 0.33 |
| Hb (g/dL) | 12.8 (10.9/14.7) | 12.8 (10.8/15.1) | 0.54 |
| MCV (fL) | 39.8 (37.5/41.9) | 40.8 (38.2/42.9) | **< 0.001** |
| MCH (pg) | 12.5 (11.7/13.2) | 12.5 (11.8/13.3) | 0.46 |
| MCHC (g/dL) | 31.7 (29.6/33.3) | 31.3 (28.8/33.2) | **0.009** |
| RDW-CV (%) | 21.0 (19.5/23.0) | 20.8 (19.2/23.1) | 0.51 |
| Leukocytes (G/L) | 13.2 (9.5/18.6) | 13.4 (8.9/20.1) | 0.85 |
| Thrombocytes (G/L) | 918 (699/1169) | 822 (506/1190) | **0.002** |

pCO_2_ = partial pressure of carbon dioxide, pO_2_ = partial pressure of oxygen, A_tot_ = non-volatile weak acids, SID_m_ = measured strong ion difference, USI = unidentified strong ions, SIG = strong ion gap, CK = creatine kinase, AST = aspartate aminotransferase, GGT = gamma glutamyltransferase, GLDH = glutamate dehydrogenase, PCV = packed cell volume, Hb = hemoglobin concentration, MCV = mean corpuscular volume, MCH = mean corpuscular hemoglobin, MCHC = mean corpuscular hemoglobin concentration, RDW-CV = red cell distribution width. ^1^ Information for pO_2_ was missing in 4 calves with a positive and 1 calf with a negative outcome. ^2^ Information for AST and GLDH was missing in 2 calves with a positive outcome. ^3^ Information was missing in 13 calves with a positive and 7 calves with a negative outcome. Information for RDW-CV was missing in 25 calves with a positive and 11 calves with a negative outcome.
